# M2-like macrophages transplantation protects against the doxorubicin-induced heart failure via mitochondrial transfer

**DOI:** 10.1186/s40824-022-00260-y

**Published:** 2022-04-11

**Authors:** Yihai Liu, Mingyue Wu, Chongxia Zhong, Biao Xu, Lina Kang

**Affiliations:** 1 Department of Cardiology, Affiliated Drum Tower Hospital, Nanjing University Medical School, 321 Zhongshan Road, 210008 Nanjing, Jiangsu China; 2 Department of Cardiology, Nanjing Drum Tower Hospital, Clinical School of Nanjing Medical University, 210008 Nanjing, Jiangsu China

**Keywords:** M2-like macrophages, Cardiotoxicity, Heart failure, Mitochondria, Cell transplantation

## Abstract

**Aims:**

The alternatively activated macrophages have shown a cardioprotective effect in heart failure. However, the effect of M2 adoptive transfer in non-ischemic heart failure is unknown. In this study, we evaluated the efficacy of M-CSF plus IL-4 induced M2-like macrophages transplantation in doxorubicin-induced cardiotoxicity.

**Methods:**

Bone marrow mononuclear cells were polarized as CCR2^+^CD206^+^ M2-like macrophages by a combination of M-CSF plus IL-4 treatment. C57BL/6 mice received a single intraperitoneal injection of doxorubicin (15 mg/kg). The treatment group were treated with M2-like macrophages (1 × 10^6 cells per mouse; i.v.) once a week for 2 weeks. After 3 weeks, we examined the percentage of resident cells and cardiac function. Furthermore, we evaluated cardiac fibrosis, cardiomyocyte apoptosis and circulating inflammatory factors. Finally, we investigated the mitochondria transfer in vitro in a direct and indirect co-culture conditions.

**Results:**

Cardiac function was significantly improved in doxorubicin-induced heart failure by adoptive transfer of M2-like macrophages. Besides, M2-like macrophages treatment attenuated cardiac fibrosis and cardiomyocyte apoptosis, as well as increased the level of circulating IL-4 and Th2 response. In vitro, M2-like macrophages could transfer mitochondria to injured cardiomyocytes in a direct and indirect way.

**Conclusions:**

In our study, adoptive transfer of M2-like macrophages could protect against the doxorubicin-induced cardiotoxicity, which may be partly attributed to mitochondria transfer. And M2-like macrophages transplantation could become a treatment for non-ischemic heart failure in the clinical practice.

**Graphical Abstract:**

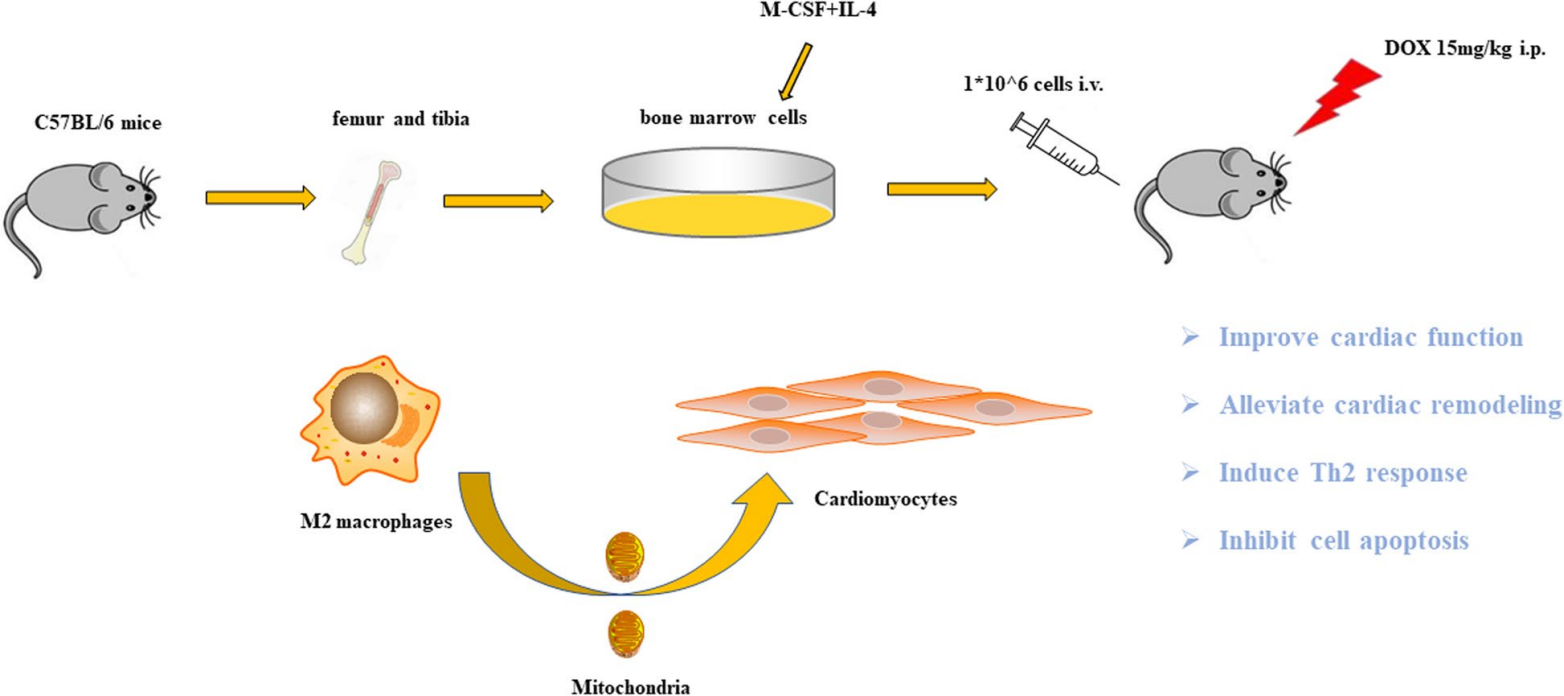

**Supplementary Information:**

The online version contains supplementary material available at 10.1186/s40824-022-00260-y.

## Introduction

Cardiovascular complications are becoming more and concerned in cancer therapies [1]. Doxorubicin (DOX), as a kind of anthracyclines, is limited by its cardiotoxicity in cancer chemotherapy, leading to progressive contractile dysfunction and ultimately heart failure (HF) [2]. A magnitude of investigations demonstrated that oxidative stress [3], chronic inflammation [4], and mitochondrial dysfunction [5] contributed to DOX-induced cardiac injury. However, no effective strategies were recommended for alleviating DOX-induced cardiotoxicity.

Macrophages are characterized by phenotypic diversity, including pro-inflammatory M1 (the classically activated M1-like) and anti-inflammatory M2 (the alternatively activated M2-like) populations [6]. Previous study showed that DOX administration significantly promotes production of the pro-inflammatory factors IL-1β, contributing to DOX-induced cardiac damage [7]. While M2 macrophages, characterized by production of IL-10, exerts cardioprotective effects in heart failure [8].

Previous reports showed that M2 adoptive transfer could prevent type 1 diabetes [9] and promoted locomotor recovery in spinal cord injury [10] by modulating inflammation response. Besides, M2-like macrophages could alleviate fibrosis [11] and promote cardiac repair post myocardial infarction [12]. Given that a low percentage of endogenous M2 activated macrophages during acute or chronic heart failure [13, 14], we speculated that M2 macrophage transplantation could effectively and clinical-applicably improve cardiac function in DOX-induced cardiotoxicity.

In this study, we cultured M2-like macrophages by treating bone-marrow-derived macrophages with M-CSF plus IL-4. Subsequently, we examined the effect of M2-like macrophage on doxorubicin-induced cardiomyopathic heart failure using an adoptive transfer method. Finally, we explored whether mitochondrial transfer mediated the beneficial effects of M2-like macrophages.

## Materials and methods

### Bone marrow derived mononuclear cells (BMDM) isolation and induction

Male C57BL/6J mice, 4 weeks old (Model Animal Research Center of Nanjing University) were euthanatized by cervical dislocation and the femur and tibia were collected. After flushed out, the red blood cells were removed using Red Blood Cell Lysis Buffer (Dakewei, China). Then the mixture was centrifuged for 5 min at 300 g and the pellet was collected. The cells were washed twice in PBS, and cultured in Dulbecco’s modified Eagle’s medium (DMEM; Gibco, USA) containing 10% fetal bovine serum (FBS, Gibco, USA) and 1% penicillin/streptomycin (P/S, Invitrogen, USA). Besides, the cells were treated with M-CSF (20 ng/ml, Peprotech) for 6 days. The medium was changed every 3 days. On the 7th day, the mature macrophages were treated with IL-4 (20 ng/ml, Peprotech, USA) for 24 h to induce the formation of M2-like macrophages. We have collected M2–like macrophages after washing for 3 times to eliminate the direct effects of IL-4 and M-CSF.

### Flow cytometry

After 7 days, cells were collected from the culture flasks by scraping and resuspended in PBS. After washed twice, cells were centrifuged at 300 g for 5 min and resuspended in 100 µl PBS. Then cells were incubated with antibodies or corresponding IgG controls for 30 min on ice.

For cardiac cell collection, the hearts were dissected, minced and enzymatically digested with type II collagenase (Sigma, USA), and passed through a 70-µm cell strainer. The following anti-mouse antibodies (eBioscience, USA) were used: anti-CCR2-APC, anti-CD206-PE, anti-CD3-FITC anti-CD4-APC, and anti-IL-4-PE. Data were analyzed with FlowJo software (Treestar, USA).

The H9c2 cardiomyocytes (Rat cardiomyocytes; American Type Culture Collection, Manassas, USA) were cultured in DMEM with 10% FBS and 1% P/S. The culture conditions contained 95% air and 5% CO_2_ at 37 ℃. An Annexin V-FITC/propidium iodide (PI) apoptosis kit (Beyotime, China) was used to analyze the apoptosis rate. The early apoptosis was gated as Annexin V^+^/PI^−^ population while late apoptosis was gated as Annexin V^+^/PI^+^ population.

### MitoTracker Red staining and carboxyfluorescein succinimidyl ester (CFSE)-fluorescent label

M2-like macrophages were stained with 200 nM MitoTracker Red (Beyotime, China) for 20 min at 37℃ and then washed twice with PBS. Pre-stained M2-like macrophages were collected and seeded for the following experiments.

H9c2 cardiomyocytes were seeded in 6-well plates and stained with 10 µM CFSE (Invitrogen, USA) for 20 min and washed twice with PBS. Pre-stained M2-like macrophages and H9c2 cardiomyocytes were co-cultured in six-well plates or Transwell plates. DAPI was stained for all cell nucleus after cells were fixed by paraformaldehyde.

Besides, condition medium of M2-like macrophages (M2-CM) was generated by collecting the medium of CFSE-stained M2-like macrophages after 24 h of culture. At the same time, medium of M2-like macrophages was filtered through a 0.22 μm syringe filter which could prevent mitochondria from entering the medium to generate mitochondria deleted medium of M2-like macrophages (Md-M2-CM).

### Animal treatment protocol

Male C57BL/6 mice (6–8 weeks old) received a single intraperitoneal injection of doxorubicin (DOX) at a dose of 15 mg/kg. One week later, a group of mice was treated with a weekly M2-like macrophages injection (1 × 10^6 cells suspended in 0.2 ml fresh DMEM/per mouse; DOX + M2 group) for two weeks via tail vein. Another group of mice was injected with DMEM accordingly (DOX group). While the third group of mice receiving no DOX was set as Sham group. Each group has 5 mice. No mortality was associated with this dosing regimen. All procedures were approved by the Institutional Animal Care and Use Committee of Nanjing Drum Tower Hospital (2019AE01062) and were in accordance with the Guide for the Care and Use of Laboratory Animals (National Academies Press, 2011).

### Transthoracic echocardiography

After 21 days, cardiac function of mice was assessed by transthoracic echocardiography using a Vevo In Vivo Micro-Imaging System (VisualSonics, Canada) under 2.0% isoflurane inhalation. Briefly, the mice were abdominally shaved, anesthetized and placed on a heat pad. The left ventricular internal systolic dimension (LVIDs) and left ventricular internal diastolic dimension (LVIDd) were measured and averaged from three consecutive cardiac cycles. And left ventricular ejection fraction (EF) and fractional shortening (FS) were calculated. EF was calculated with Simpson method [15] and FS was calculated as [(LVIDd-LVIDs)/LVIDs] x100%.

### Histological analysis

Hearts from each group were washed in PBS and fixed in 4% formalin, and embedded in paraffin and sliced into 5 μm-thick tissue sections. Heart slices were stained with haematoxylin-eosin staining (HE) and observed under the light microscope (Olympus, Japan). Three HE-stained heart sections from each group were examined for the evaluation of cytoplasmic vacuolization. The cardiomyocytes with vacuoles were counted and normalized to all cardiomyocytes in the section. To observe collagen deposition, the heart sections were stained with Masson trichrome. To observe the cross-sectional area, the sections were dewaxed, rehydrated and subjected to wheat germ agglutinin-FITC for 1 h. Sections were further stained with DAPI. After washed three times, the slides were mounted with a fluorescent microscopy (Keyence, UK). The semi-quantification results were obtained using Image J (NIH, USA).

### Western blotting

Heart tissues were homogenized and protein concentrations were measured using a BCA kit (Thermo, USA). Total protein lysates were separated by SDS-PAGE and transferred to PVDF membranes (Millipore, USA). The membranes were incubated with the corresponding primary antibodies overnight at 4 ℃ and then incubated with goat anti-rabbit lgG. The western blot bands were detected using ECL kit (Keygene; China). The membrane was first immunoblotted with the phosphorated protein and then washed, and immunoblotted with the total protein. The protein expression levels were normalized to GAPDH levels. Following antibodies (Abcam, USA) were used: cleaved- caspase 3, AKT, p-AKT, ERK1/2, and p-ERK1/2. The relative expression of protein was averaged using Image J.

### Immunofluorescence staining

Heart cryosections from mice injected with CFSE-labelled M2-like macrophages or MitoTracker Red-labelled M2-like macrophages were stained with the DAPI to visualize the nucleus.

To show the percentage of Th2 cells within myocardium, heart sections were incubated overnight with GATA3 (CST, USA). After washing 3 times, fluorescent secondary antibodies were added.

Every section was observed using the fluorescence microscopy (Olympus, Japan) and analyzed using Image J (NIH, USA).

Cardiac apoptosis was detected by the TdT-mediated dUTP nick end labelling (TUNEL) apoptosis assay kit (Beyotime, China) according to the protocol.

### Enzyme linked immunosorbent assay (ELISA)

After 21 days, the mouse serum was collected. The level of inflammatory factors were measured with the ELISA Kits (Servicebio, China) according to the manufacture’s protocol. The optical densities of the samples were detected using a microplate reader (Bioteck, USA) at a wavelength of 450 nm.

### Isolation of mitochondria from M2-like macrophages and cell culture

Mitochondria were isolated from cultured M2-like macrophages using a Mitochondria Isolation Kit (Beyotime, China) according to the instructions. The freshy isolated mitochondria were used in the following study.

H9c2 cardiomyocytes were cultured at 37℃ with 5% CO_2_ in DMEM supplementing with 10% FBS and 1% P/S. To mimic cardiac injury *in vivo*, H9c2 cardiomyocytes were treated with 1 µM of doxorubicin (DOX group; Sigma, USA) for 24 h. Treatment group was added with mitochondria (from 1 × 10^6 cells) plus doxorubicin (DOX + Mito group) for 24 h.

### Lactate dehydrogenase (LDH) release assay

After cultured for 24 h, the culture supernatants of H9c2 cardiomyocytes were collected to detect the level of LDH release using a LDH assay kit (Beyotime, China) according to the manufacturer’s instructions. Experiments were performed at least in triplicate. Data were expressed as percentages of control values.

Data are presented as mean ± standard deviation. Analysis was performed using Prism 7 software (GraphPad, USA). Between-group differences were assessed by Student’s t-test or one-way analysis of variance with Bonferroni post-hoc test. The statistical significance level was set at *P* < 0.05.

## Results

### M-CSF + IL-4 treatment was effective to produce M2-like macrophages

**Fig. 1:**
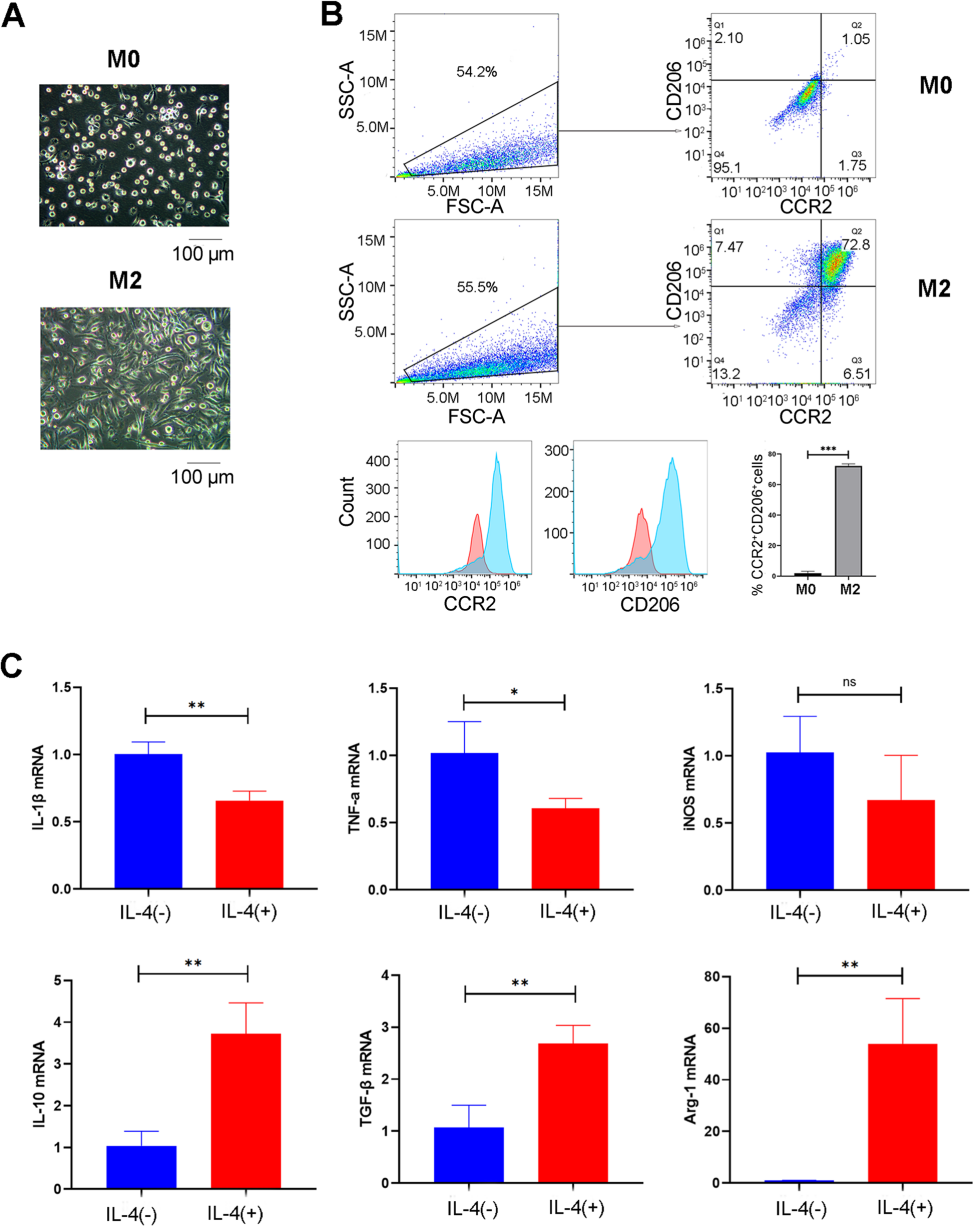
Production of M2-like macrophages by M-CSF + IL-4 treatment. **A** The cell morphology was shown under the light microscopy. M0 macrophages (without M-CSF plus IL-4 treatment) were used as controls. Scale bar 100 μm. **B** The surface expression of CCR2 and CD206 was measured using flow cytometry. Below was the fluorescent intensity of CCR2 and CD206, and the quantification results. **C** The gene expression of M1- and M2-related markers, including IL-1β, TNF-α, iNOS and IL-10, TGF-β, Arg-1, were measured by qPCR. (Data were presented as mean ± SD. ***p < 0.001. N = 3 per group)

To produce M2 macrophage, mouse BM-MNCs were treated with M-CSF for 6 days, followed by IL-4 for 1 day. Under a microscope, we observed that M2-like macrophages had a typical spindle shape compared with naïve M0 macrophage with a round appearance (Fig. 1A). In addition, the results of flow cytometry (Fig. 1B) demonstrated that induced macrophages highly expressed macrophage (CCR2) and M2 macrophage (CD206) surface markers (15.8- and 79.4-fold changes respectively). In summary, after 7 days of in vitro culture with 6-day M-CSF treatment followed by 1-day of IL-4 treatment, over 70% of the BM-MNCs were polarized into M2-like macrophages. In addition, qPCR results (Fig. 1C) confirmed that M2-like macrophages had a higher expression of M2-related markers (IL-10, TGF-β和Arg-1) while a lower expression of M1-related markers (IL-1β, IL-6 和TNF-α).

### Adoptive transfer of M2-like macrophages prevents doxorubicin-induced left ventricular dysfunction

First, we tested the protective effects of M2-like macrophages on heart damage by DOX. A group of mice received a single injection of DOX (15 mg/kg, i.p.). While a group of mice receiving no DOX was regarded as Sham group. After 7 days, the echocardiography results suggested that the heart was already injured (Supplementary Table 1). Then we treated DOX-injured mice with DMEM (DOX group) or M2-like macrophages (1 × 10^6 cells/per mouse; DOX + M2 group) once a week for two weeks.

**Fig. 2:**
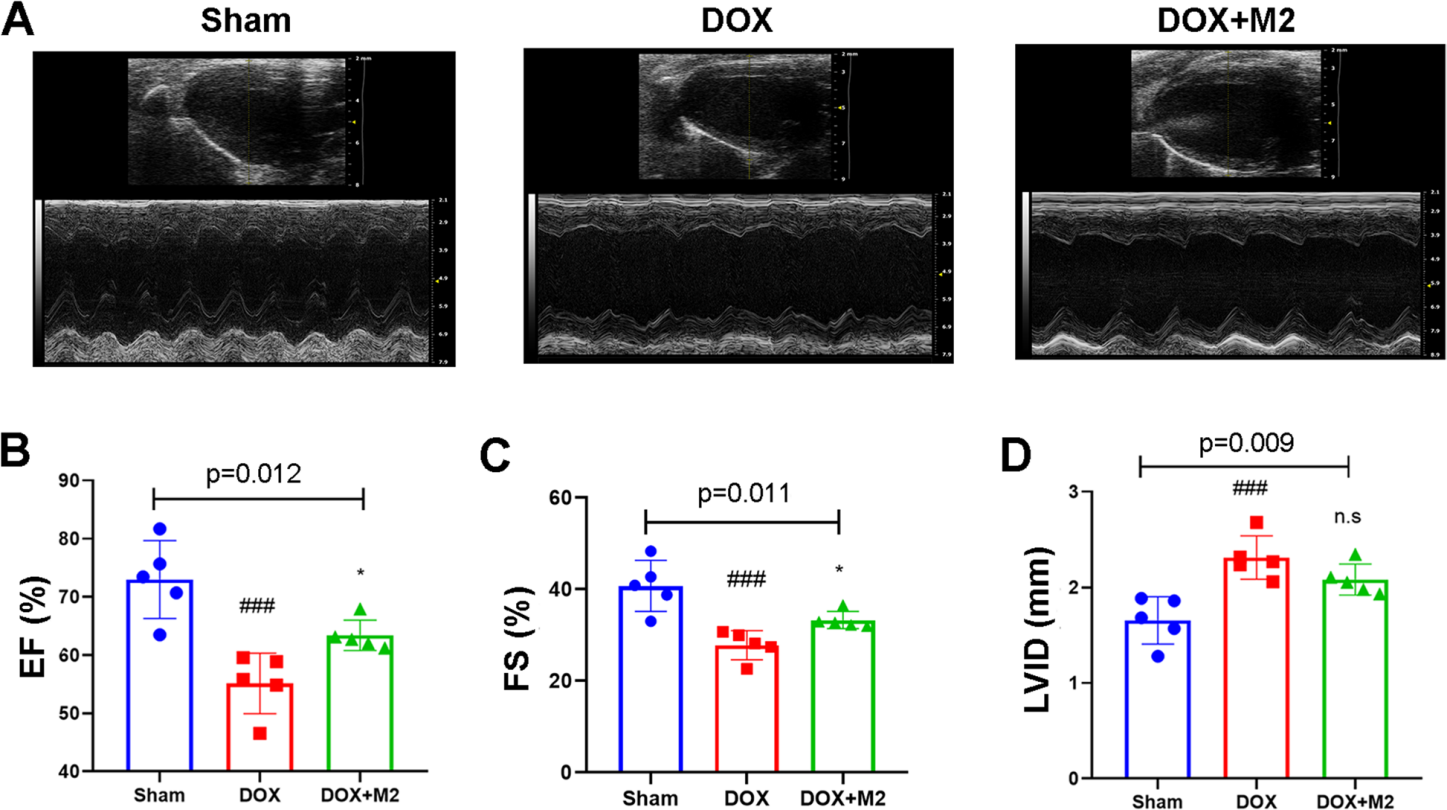
Cardiac function between groups were measured using transthoracic echocardiography between groups. **A** Representative echocardiography images between groups. **B** Ejection fraction (EF), **C** Fractional shortening (FS) and **D** left ventricular internal dimension (LVID) at systole were measure among groups. (Data were presented as mean ± SD. ***P < 0.001 vs. Sham; #*P* < 0.05 vs. DOX. N = 5 per group, One-way ANOVA)

After 21 days, transthoracic echocardiography (Fig. 2A) *in vivo* showed impaired systolic function: EF was 55.1 ± 5.2% in DOX group vs. 72.9 ± 6.7% in Sham group (*P* < 0.001); FS was 27.8 ± 3.2% in DOX group vs. 40.7 ± 5.6% in Sham group (*P* < 0.001) (Fig. 2B, C). Intriguingly, adoptive transfer of M2-like macrophages resulted in a significant improvement of left ventricular dysfunction compared with DOX group: EF was 63.4 ± 2.6% (*P* = 0.025); FS was 33.3 ± 1.9% (*p* = 0.044). Even though, M2-like macrophages could not fully reverse the DOX effect compared with control group (*P* = 0.012 in EF; *P* = 0.011 in FS). However, M2 macrophage had no significant effect on increased LVID induced by DOX (Fig. 2D).

### M2-like macrophages transplantation prevent doxorubicin-induced cardiac remodeling and injury

**Fig. 3:**
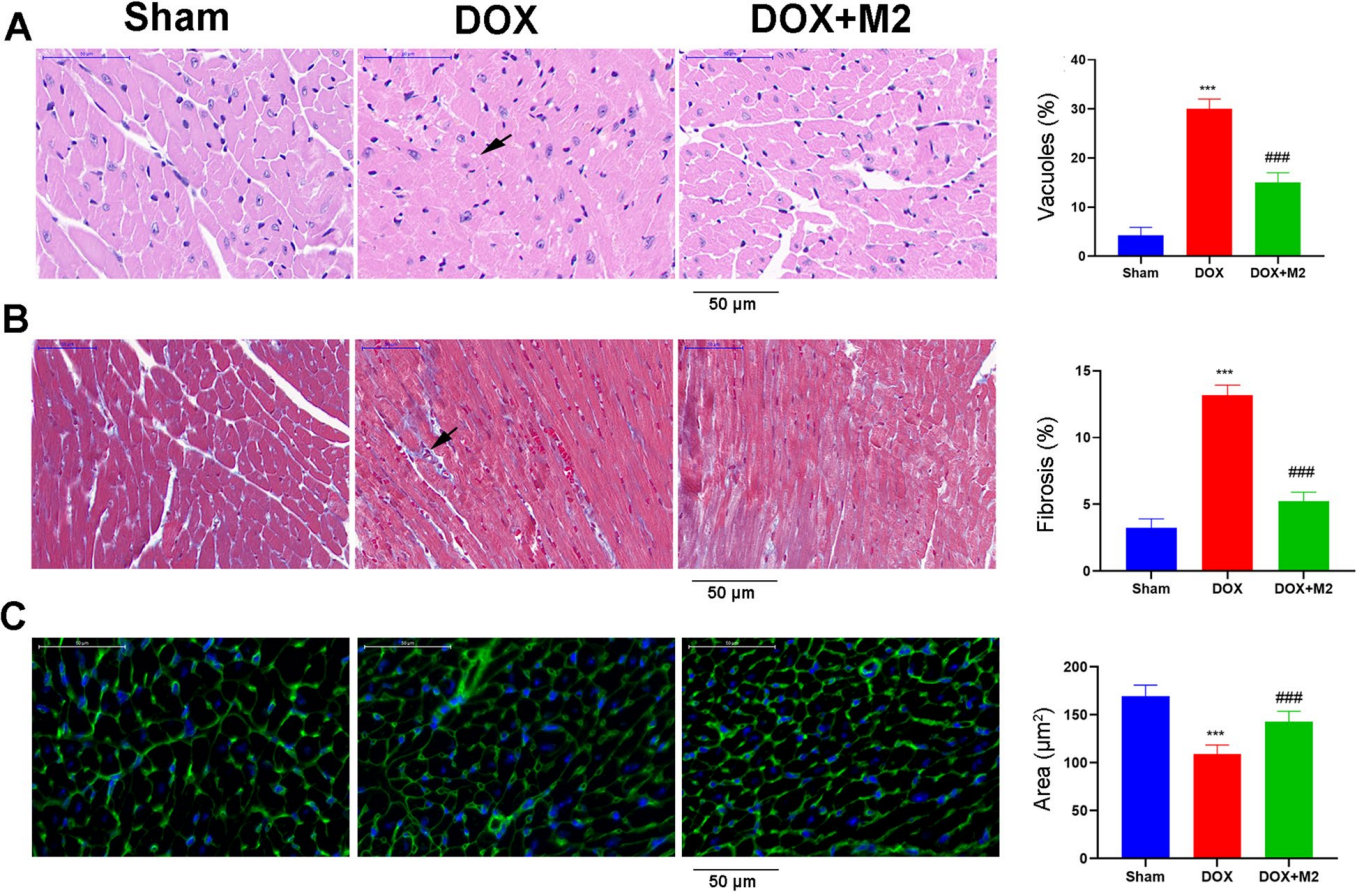
Histological staining was used to evaluate cardiac remodeling among groups. **A** HE staining for quantification of cardiomyocyte vacuoles. **B** Masson staining for quantification of interstitial fibrosis. **C** Wheat germ agglutinin staining for measurement of cell size. The arrowheads showed corresponding alterations. Three microscopic fields were analyzed per animal. (Data were presented as mean ± SD. ***P < 0.001 vs. Sham; ###*P* < 0.001 vs. DOX. *N* = 5 per group)

Next, we explored the effect of M2-like macrophages on some hallmarks of DOX cardiotoxicity. Cytoplasmic vacuolization, an adverse architectural alteration commonly described in DOX cardiotoxicity. HE staining showed a significant increase of cardiomyocyte vacuoles in DOX hearts (Fig. 3A), which was counteracted by M2 transfer. Similar, DOX caused smaller cardiomyocyte size (Fig. 3B) and more interstitial fibrosis (Fig. 3C), which were reversed by M2 treatment. These results suggested that M2 transplantation prevented cardiac remodeling.

### Adoptive transfer of M2-like macrophages increases the level of circulating IL-4

**Fig. 4:**
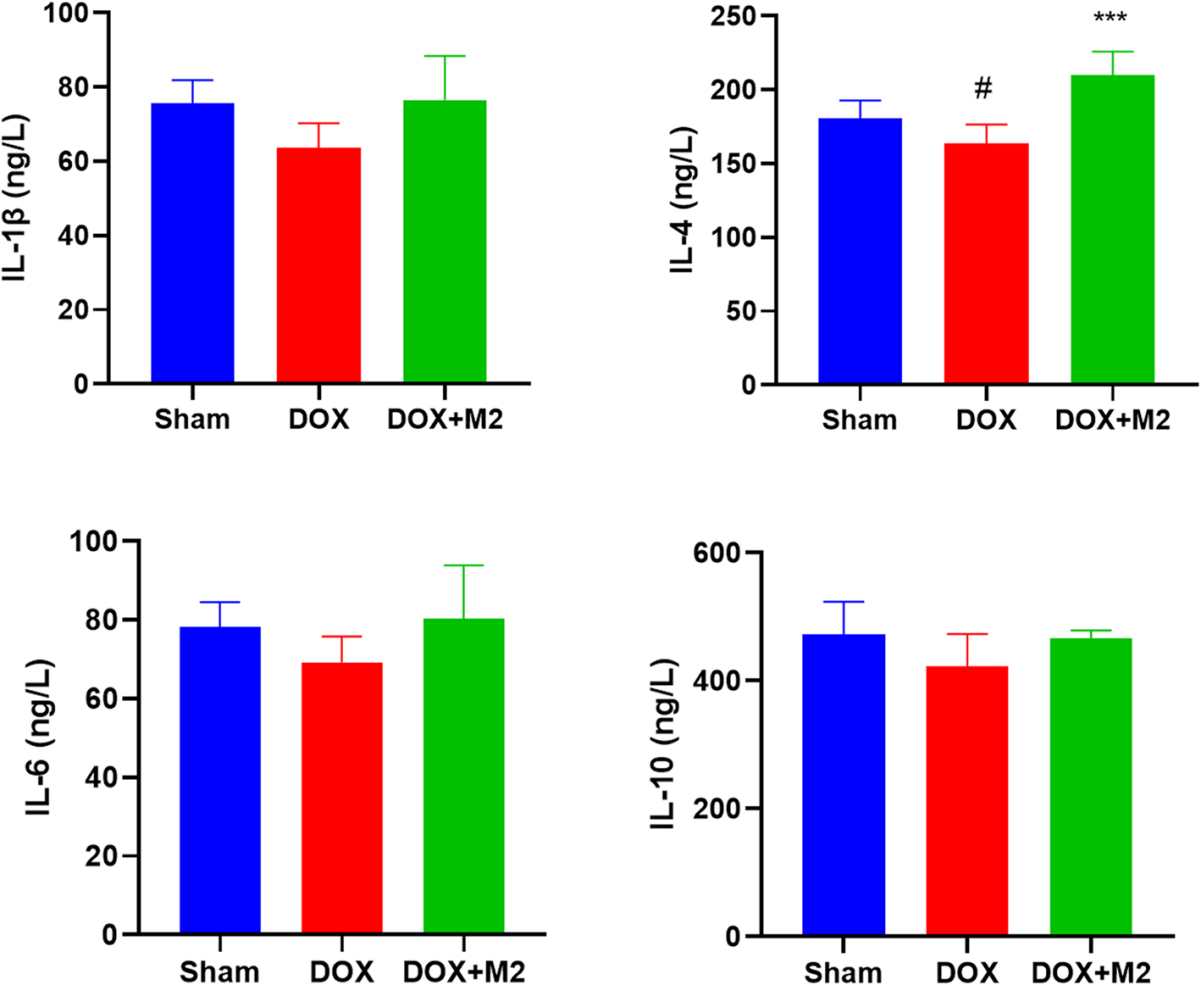
The circulating inflammatory factors were measured by enzyme-linked immunosorbent assay after 3 weeks. The levels of **A** IL-1β, **B** IL-4, **C** IL-6, and **D** IL-10 among groups. (Data were presented as mean ± SD. *P < 0.05 vs. Sham; ###*P* < 0.001 vs. DOX. *N* = 5 per group)

To investigate the effect of M2 macrophage infusion on the inflammatory response, we measured the level of inflammatory cytokines in mouse serum after 3 weeks. As shown in Fig. 4, DOX decreased the level of circulating IL-4, which was rescued by M2 macrophage (209.7 ± 16.3 ng/L in DOX + M2 group vs. 163.6 ± 13.0 ng/L in DOX group, *P* < 0.001). However, in terms of IL-1β, IL-6 and IL-10, there was no statistical difference among groups.

### M2-like macrophages transplantation induced Th2 response

**Fig. 5:**
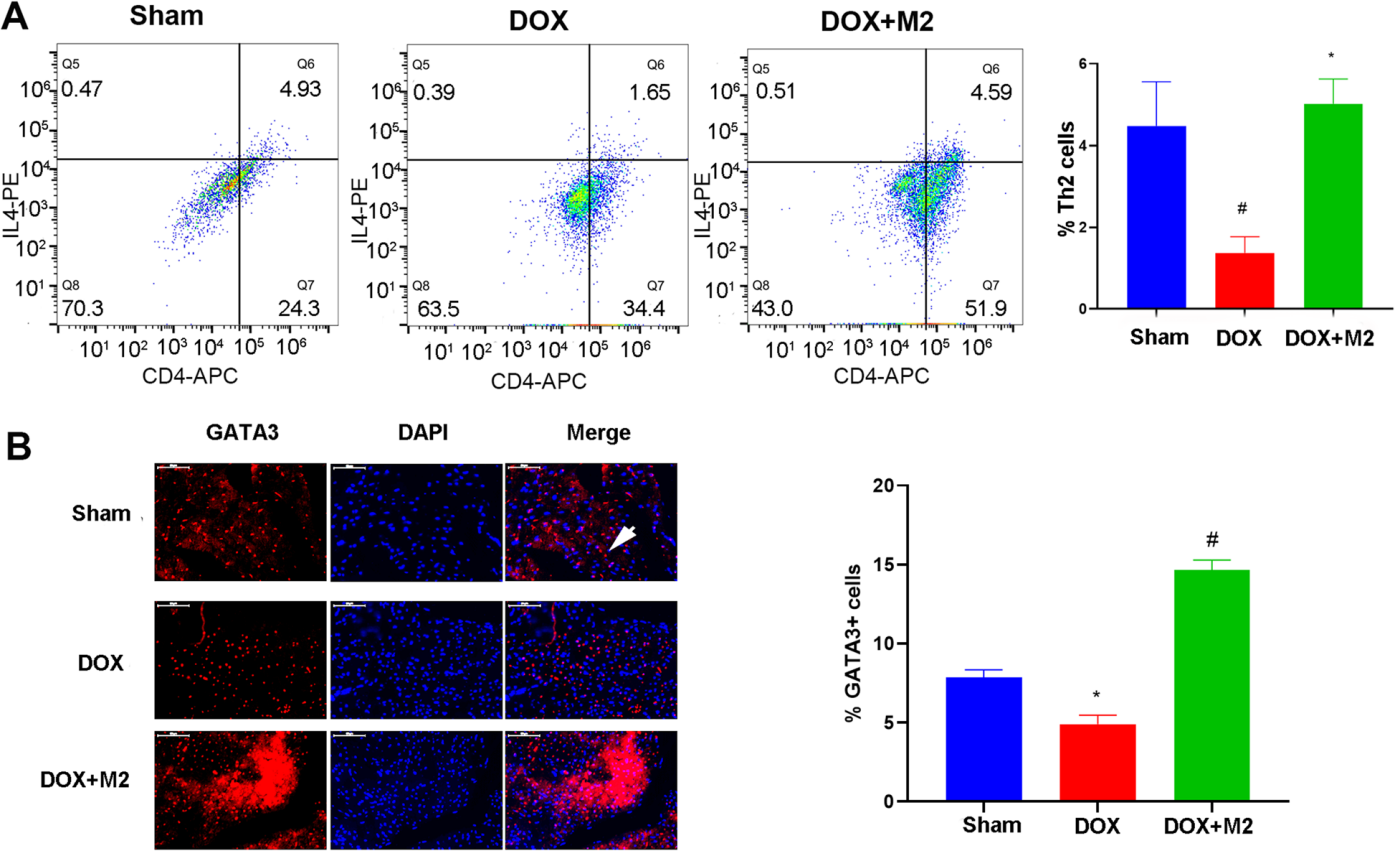
M2-like macrophages induced the Th2 response within myocardium. **A** The CD4^+^IL-4^+^Th2 cells were measured using flow cytometry and were quantified as the percentage of cardiac cells. **B** The presence of GATA3^+^ cells within the hearts were observed by immunofluorescence staining and were quantified as the percentage of cardiac cells. The arrowhead showed the GATA3^+^ cells. (Data were presented as mean ± SD. *P < 0.05 vs. Sham; #*P* < 0.05, ##*P* < 0.01 vs. DOX. *N* = 5 per group)

It was acknowledged that M2 macrophage could secrete immune cytokines to induce Th2 response. Consistently, the results of flow cytometry showed that M2 macrophage increased the percentage of CD4^+^IL-4^+^Th2 cells (Fig. 5 A). In addition, the results of immunofluorescence staining demonstrated that a higher percentage of GATA3^+^cells, which was a specific transcription factor of Th2 cells and used to label Th2 cells [10, 16], existed in hearts of DOX + M2 group compared with that of DOX group (Fig. 5B).

### M2-like macrophages inhibit cardiomyocyte apoptosis independent of AKT and ERK pathway

**Fig. 6:**
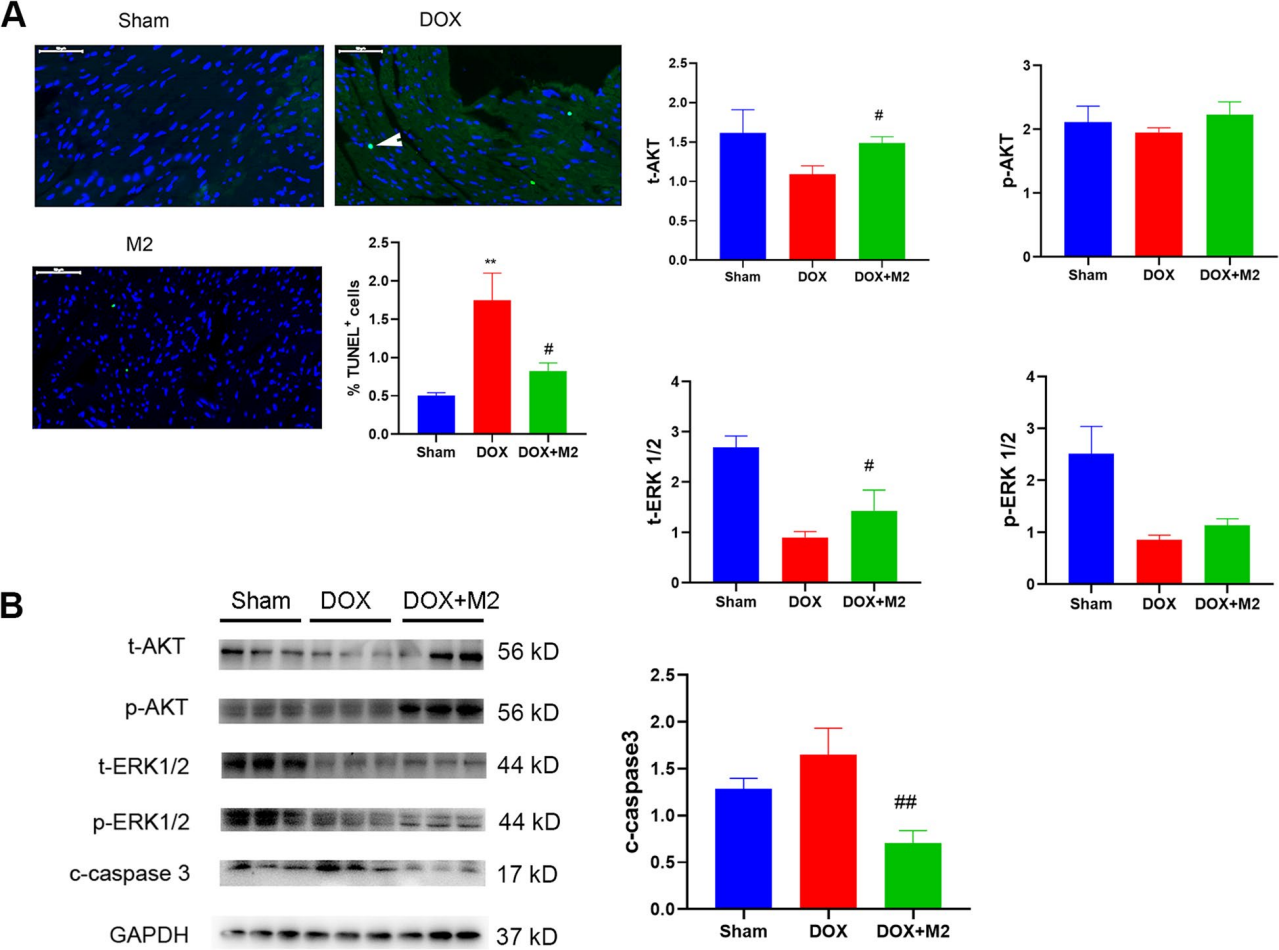
M2-like macrophages inhibited cardiomyocyte apoptosis. **A** The TUNEL staining of apoptotic cardiac cells. The arrowhead showed the TUNEL-positive cell. **B** The immunoblotting results among groups and the gray value of t-AKT, p-AKT, t-ERK 1/2, p-ERK 1/2, and c-caspase 3 from up-left to down-right. (Data were presented as mean ± SD. *P < 0.05, **P < 0.01 vs. Sham; #*P* < 0.05, ##*P* < 0.01 vs. DOX. *N* = 5 per group)

Cardiomyocyte apoptosis contributed to adverse remodeling in DOX-induced cardiac injury. As shown in Fig. 6A, the results of TUNEL staining showed that DOX caused cardiomyocyte apoptosis, which was prevented by adoptive transfer of M2-like macrophages. In addition, immunoblotting results (Fig. 6B) demonstrated that the level of c-caspase 3 was decreased in DOX + M2 group compared with DOX group. Meanwhile, we observed that AKT and ERK1/2 were upregulated while p-AKT and p-ERK1/2 were not significantly changed upon M2 macrophage treatment. These results demonstrated that M2 macrophage could inhibit cardiomyocyte apoptosis independent of AKT and ERK pathway.

### M2-like macrophages could transfer mitochondria to the cardiomyocytes

**Fig. 7:**
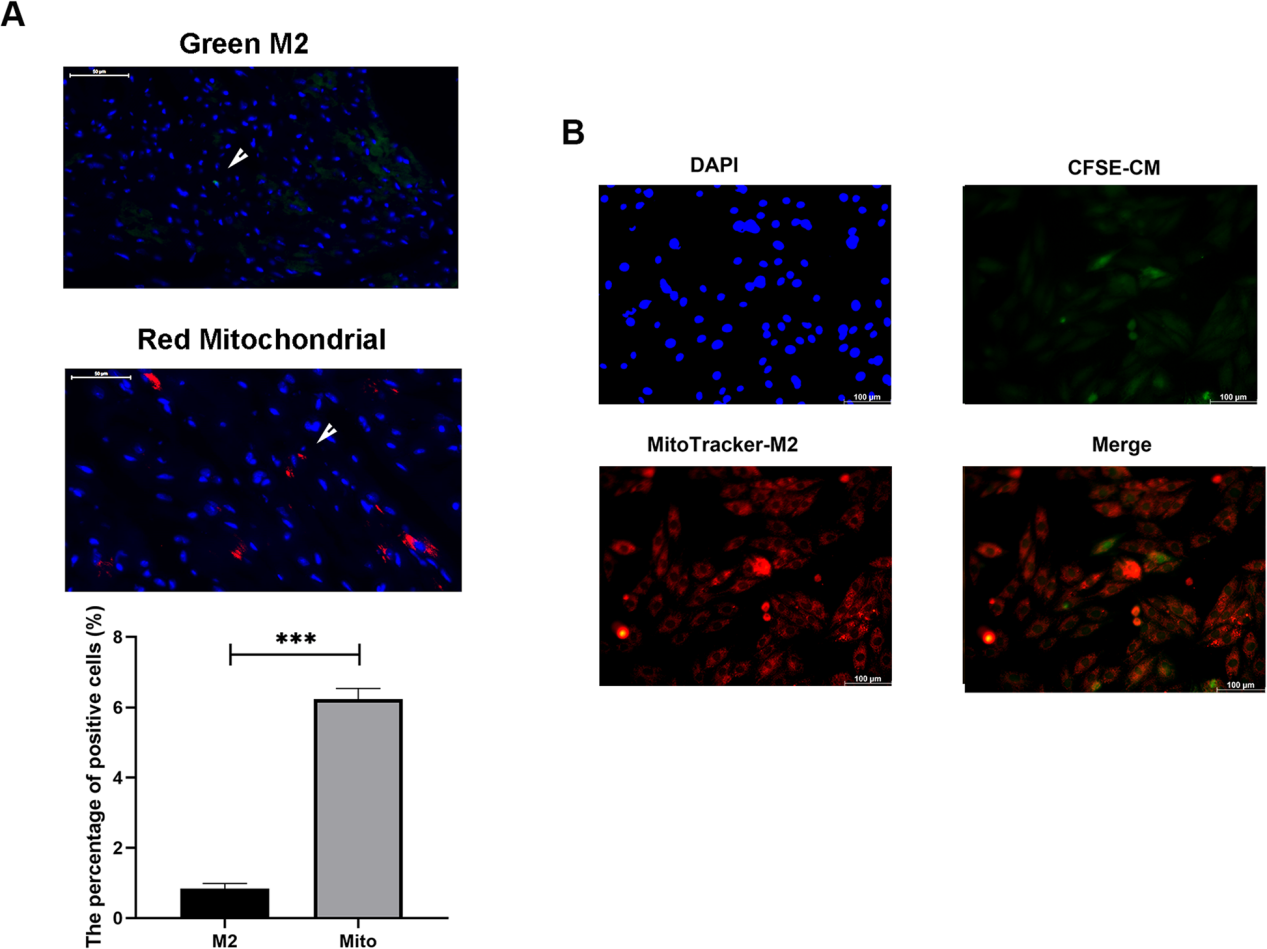
M2-like macrophages could transfer mitochondria to cardiomyocytes in vivo and in vitro. **A** The green fluorescence showed the presence of M2-like macrophages within the myocardium while the red positive mitochondria were localized inside the myocardium. The quantitative results were shown as the histogram at the left-bottom. **B** Red fluorescence dots (mitochondria) were incorporated into the body of CSFE-labelled cardiomyocytes. (Data were presented as mean ± SD. ***P < 0.001. *N* = 3 per group)

To investigated the cell engraftment within myocardium, we labelled M2-like macrophages with CFSE and treated the mice via tail injection. After 24 h, we sacrificed the mice and sliced the hearts. The green fluorescence showed that very few M2-like macrophages were resident within the myocardium (Fig. 7A). Furthermore, mice were injected with MitoTracker red pre-stained M2-like macrophages to show the distribution of M2-like macrophages derived mitochondrial. The red positive mitochondria were localized inside the myocardium, which meant that M2-like macrophages could transfer mitochondrial to the cardiomyocyte *in vivo*.

What’s more, we confirmed the transfer of mitochondrial in direct co-culture conditions *in vitro*. Pre-stained H9c2 cardiomyocyte (CFSE) and M2-like macrophages (MitoTracker Red) were co-cultured in six-well plate for 24 h followed by fluorescence microscopy. Figure 7B showed that MitoTracker Red labelled mitochondria were transferred from M2-like macrophages to H9c2 cardiomyocytes as evidenced by red fluorescence dots incorporated into the body of CSFE-labelled cardiomyocytes.

### Transfer of Mitochondria from M2 macrophage to cardiomyocyte in an indirect co-culture condition

**Fig. 8:**
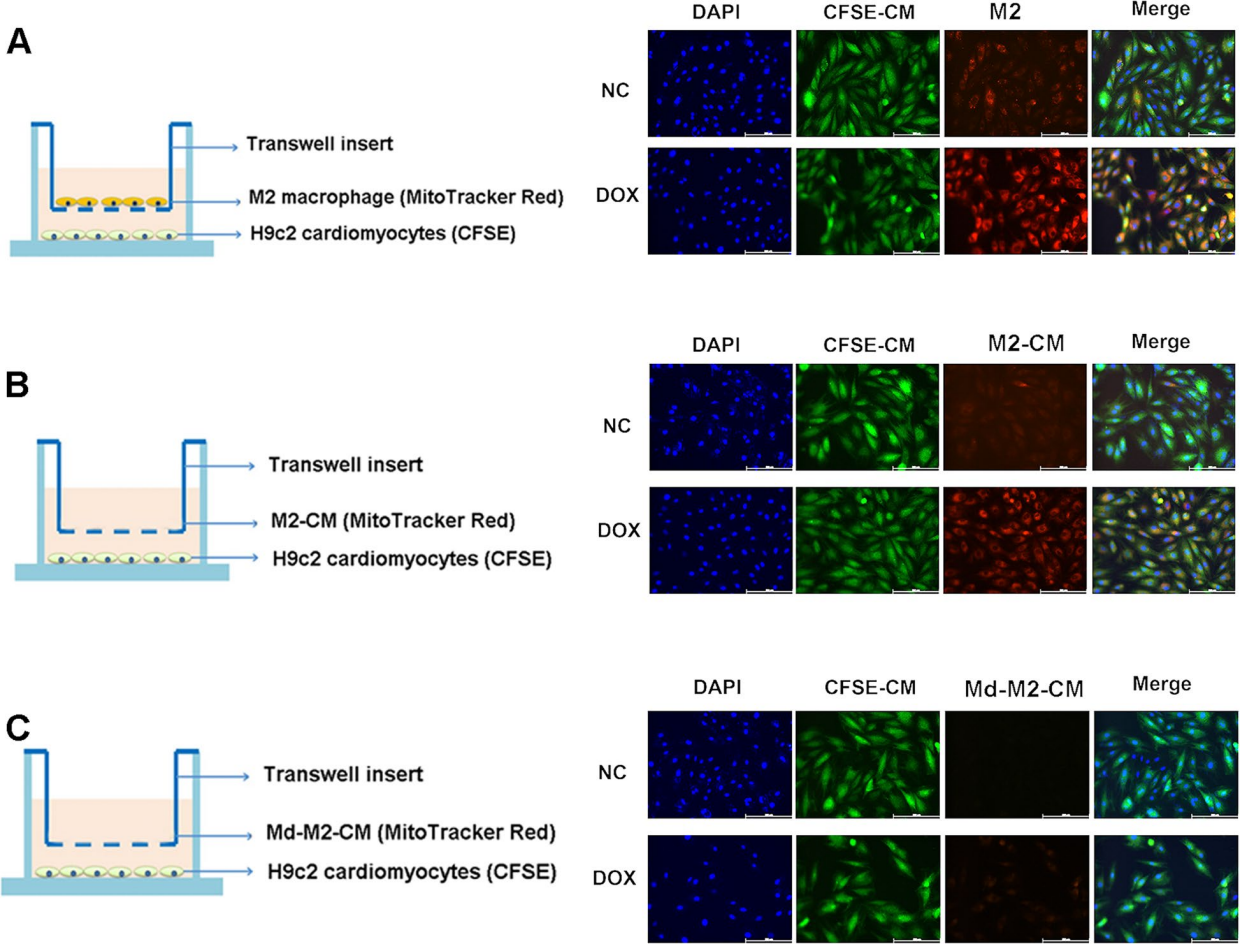
Transfer of Mitochondria from M2 macrophage to cardiomyocyte in a transwell plate (8 μm pore). **A** Up: M2 macrophage derived mitochondrial were located inside the body of cardiomyocytes. **B** Middle: M2 condition medium derived mitochondrial were internalized into the body of cardiomyocytes. **C** Down: No mitochondria were taken by cardiomyocytes using mitochondria-depleted condition medium of M2-like macrophages

Meanwhile, we considered whether paracrine mechanism may also play a role in mitochondria transfer. Therefore, firstly, the pre-stained M2-like macrophages were co-cultured with H9c2 cardiomyocytes in a transwell plate (8 μm pore) for 24 h (Fig. 8 Up). Fluorescent microscopy observation showed M2-like macrophages derived mitochondrial were located inside the body of cardiomyocytes. Besides, there were more mitochondria incorporated into DOX-injured cardiomyocytes. Secondly, we cultured cardiomyocytes in the condition medium of M2-like macrophages (M2-CM) (Fig. 8 Middle). Fluorescent microscopy observation showed MitoTracker red labelled mitochondrial were internalized into the body of cardiomyocytes. And DOX-injured cardiomyocytes could take up more percentage of mitochondrial. Thirdly, we culture cardiomyocytes in mitochondria-depleted condition medium of M2-like macrophages (Md-M2-CM) (Fig. 8 Down). Fluorescent microscopy observation showed no MitoTracker red labelled mitochondria were in the body of cardiomyocytes, neither in PBS-treated cardiomyocytes or DOX-treated cardiomyocytes.

### Mitochondria internalization alleviated the cell stress induced by DOX

**Fig. 9:**
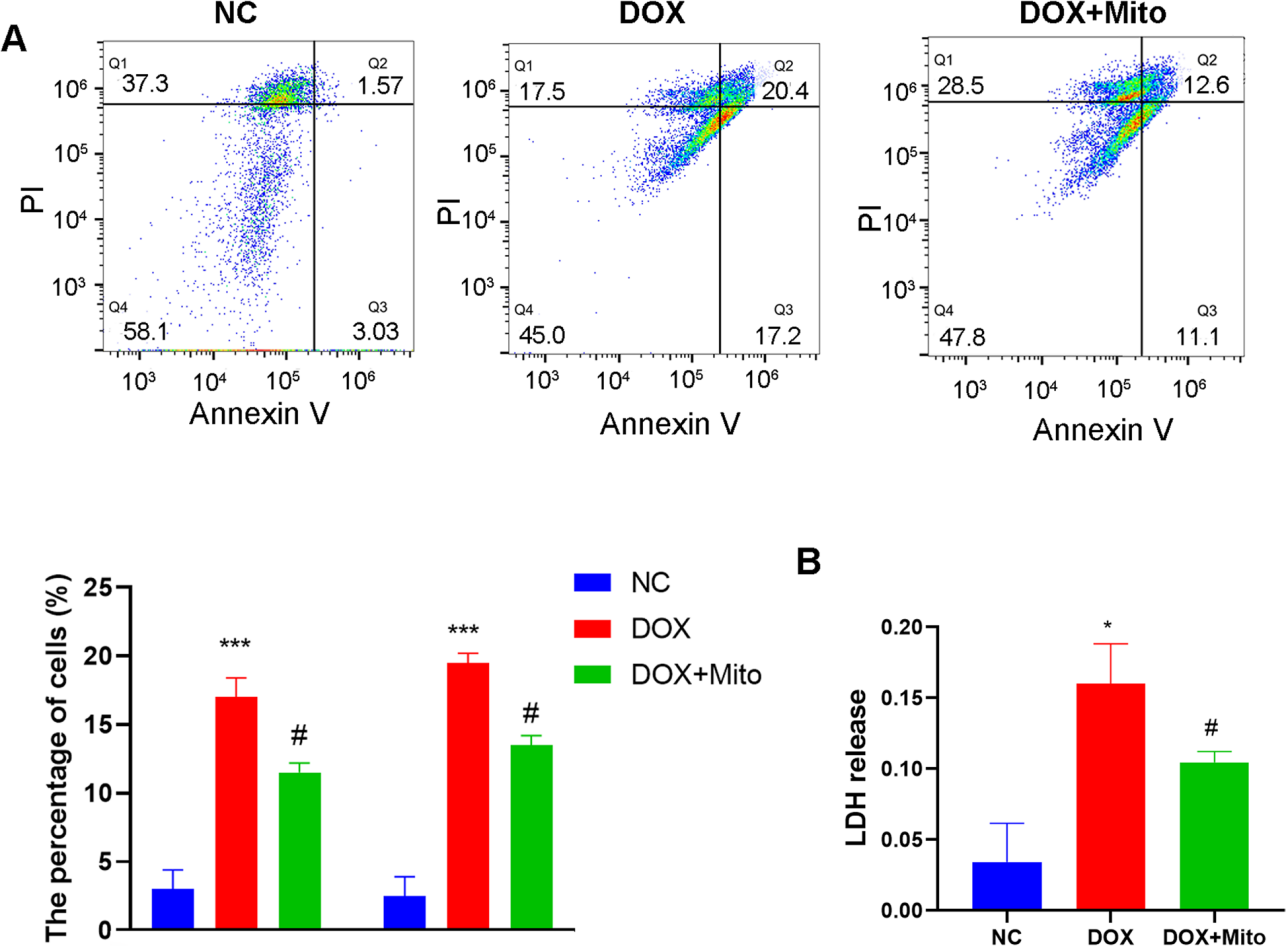
Mitochondria internalization alleviated the cell stress induced by DOX. **A** Mitochondria treatment decreased the LDH release level. **B** Mitochondria treatment decreased the percentage of Annexin V + PI- and Annexin V + PI + cells. (Data were presented as mean ± SD. *P < 0.05, ***P < 0.001 vs. Sham; #*P* < 0.05 vs. DOX. *N* = 3 per group)

To explore the beneficial effects of M2-like macrophages, we isolated the intact mitochondria from M2-like macrophages and cultured them with injured cardiomyocytes. Our results showed that mitochondria (from 1 × 10^6 M2-like macrophages/ well) treatment decreased the LDH release level, which was increased upon DOX treatment (Fig. 9A). Besides, flow cytometry results showed that DOX lead to more percentage of Annexin V^+^PI^−^ and Annexin V^+^PI^+^ cells, which was prevented by mitochondria treatment (Fig. 9B). These results showed that mitochondria treatment could inhibit cell apoptosis induced by DOX.

## Discussion

In this study, we showed that adoptive transfer of M2-like macrophages had beneficial effects on non-ischemic heart failure, and the beneficial effects could be related to the mitochondria transfer. Specially, M2-like macrophages transplantation upregulated circulating IL-4 levels and induced cardiac Th2 immune response, as well as prevented from cardiomyocytes apoptosis. In vitro, M2-like macrophages could transfer mitochondria to cardiomyocytes in a direct and indirect co-culture method [9].

Previous studies have showed a therapeutic effect of macrophage transplantation, whether originating from embryonic stem cells, induced pluripotent stem cells, or bone marrow mononuclear cells, on various organ injury, including liver [17], lung [11] and heart [18]. Using a combination of M-CSF and IL-4 treatment, we efficiently induced reparative M2-like macrophages in vitro. In ischemic heart failure, M2 macrophages transplantation enhanced cardiac repair, achieving a superior therapeutic efficacy compared to bone marrow mononuclear cells [12]. In consistent with previous studies, our results also showed that M2-like macrophages transplantation alleviated cardiac fibrosis and systolic dysfunction. Previous study found that Th1 polarization existed in heart failure, while atorvastatin exhibited beneficial effects by regulating Th1/Th2 response [19]. Immune modulation therapy improved cardiac function of patients with chronic cardiac insufficiency accompanied by increased expression of Th2 and GATA-3 mRNA [20]. AKT and ERK signaling pathways were involved in cell survival and apoptosis upon stress [21]. Previous studies have found that upregulation of AKT [22] and ERK [23] promoted the survival of cardiac myocytes. In consistent with that, we also found that M2-like macrophages induced Th2 cell activation and inhibited cardiomyocyte apoptosis, contributing to a cardioprotective role.

Regarding the possible underlying mechanism for improved cardiac function after M2-like macrophages transplantation, several paracrine factors released from transplanted cells have been suggested. M2 macrophages were reported to secrete enzymes [24, 25] cytokines [9, 12], peptides [26], exosomes [27] which could mediate its beneficial effects. What’s more, local M2-like macrophages participated in some signal pathways, including NF-kB [28], and RIG-I [29] and regulated Treg and Th17 cell responses [30]. Similarly, our results found M2-like macrophages transplantation induced Th2 response as evidenced by more filtration of IL4^+^ or GATA3^+^CD4^+^ T cells. Surprisingly, we found that mitochondria transfer was a novel mechanism for the beneficial effect of M2-like macrophages.

Cardiomyocytes harbor a network of mitochondria for a large energy demand. The structural and function integrity is a perquisite for mitochondria to supporting cardiac function. However, doxorubicin-induced cardiac injury was characterized by perturbed mitochondrial function, including mitochondria autophagy [31], mitochondria fission/fusion [32] and oxidative phosphorylation [33]. Mitochondrial could mediate the cell communication [34], thereby replacing the compromised mitochondria in host cells [35], and maintaining necessary function [36]. For example, mesenchymal stem cells could promote the oxidative phosphorylation of macrophages through extracellular vesicle-mediated mitochondria transfer [37]. Besides, they can transfer mitochondria to lymphoblastic leukemia cells [38] or hypoxic neurons [39] to prevent from oxidative stress. A study found that cardiac macrophages could phagocytize the mitochondria released from injury cardiomyocytes, contributing to cardiac repair [40]. In our study, we further found that mitochondria could transfer from M2-like macrophages to compromised cardiomyocytes and promote cell survival under stress.

Limitation existed in our study. Firstly, previous studies reported that extracellular mitochondrial could be packed in exosomes [41, 42], or as whole organelles [35, 43]. We speculated that they may be transferred via exosomes. The mechanism of mitochondrial transfer should be investigated in the future. Secondly, there are more than 70% of M2-macrophages and some M0 or M1 macrophages in the population. It would be better to isolate and purify M2 macrophages as a treatment method in the future.

## Conclusions

In summary, we found that adoptive transfer M2-like macrophages could protect against the doxorubicin-induced cardiotoxicity, which may be partly attributed to mitochondria transfer.

## Supplementary Information

**Additional file 1.**

## Data Availability

All data supporting our conclusions could be available from the corresponding author.
