# Supplementary material for: M2-like macrophages transplantation protects against the doxorubicin-induced heart failure via mitochondrial transfer
Source: Biomater Res. 2022 Apr 11;26:14. doi: 10.1186/s40824-022-00260-y (PMC8996664; doi:10.1186/s40824-022-00260-y)
Supplement: Supplementary file 1 — Additional file 1. [file 40824_2022_260_MOESM1_ESM.docx]

**Supplementary Table 1.** The echocardiography results of DOX-injured mice after one week since the single injection. P was calculated using the Unpaired t-test.

|  | Sham | DOX | P |
| --- | --- | --- | --- |
| **IVS;d** | 0.73 ±0.04 | 0.66 ±0.03 | 0.005 |
| **IVS;s** | 1.13 ±0.07 | 1.00 ±0.05 | 0.004 |
| **LVIDd** | 3.55 ±0.16 | 3.76 ±0.33 | 0.191 |
| **LVIDs** | 2.34 ±0.14 | 2.70 ±0.32 | 0.033 |
| **LVPW;d** | 0.75 ±0.05 | 0.65 ±0.01 | 0.001 |
| **LVPW;s** | 1.19 ±0.07 | 0.98 ±0.05 | <0.001 |
| **EF** | 63.98 ±1.72 | 55.56 ±4.74 | 0.002 |
| **FS** | 34.05 ±1.18 | 28.44 ±3.04 | 0.001 |
